# Muscle Strength and Functional Performance as Predictors of Metabolic and Body Composition Improvement in Anorexia Nervosa

**DOI:** 10.3390/nu18040642

**Published:** 2026-02-16

**Authors:** Eugenia Dozio, Sofia Gritti, Lorenzo Niego, Elisa Sartorello, Edoardo Scuttari, Gianluca Tori, Arianna Ruggiero, Letizia Galasso, Lucia Castelli, Angela Montaruli, Eliana Roveda, Rina Giuseppa Russo, Andrea Caumo, Ileana Terruzzi

**Affiliations:** 1 Villa Miralago, Center for the Treatment of Eating Disorders, Cuasso al Monte, 21050 Varese, VA, Italy; eugenia.dozio@villamiralago.it (E.D.); scuttarie@gmail.com (E.S.); rina.russo@villamiralago.it (R.G.R.); 2 Department of Medicine and Technological Innovation (DIMIT), University of Insubria, 21100 Varese, VA, Italy; 3 Biology Applied to the Sciences of Nutrition, Università degli Studi di Milano, 20133 Milano, MI, Italy; sofia.gritti1@studenti.unimi.it (S.G.); niegolorenzo@gmail.com (L.N.); 4 Department of Biomedical Sciences for Health, Università degli Studi di Milano, 20133 Milano, MI, Italy; elisa.sartorello@studenti.unimi.it (E.S.); letizia.galasso@unimi.it (L.G.); lucia.castelli@unimi.it (L.C.); angela.montaruli@unimi.it (A.M.); eliana.roveda@unimi.it (E.R.); andrea.caumo@unimi.it (A.C.); 5 Alkemy S.p.A., 20124 Milano, MI, Italy; gianluca.tori@alkemy.com; 6 Unisalute S.p.A., Via Larga, 8, 40138 Bologna, BO, Italy; ariannaruggiero024@gmail.com

**Keywords:** anorexia nervosa, muscle strength, functional performance, body composition, metabolic status

## Abstract

**Background/Objectives**: Anorexia nervosa (AN) is a severe psychiatric disorder characterized by energy restriction and associated with profound metabolic and body composition alterations. The loss of Body Cell Mass (BCM) leads to impaired muscle strength and functional capacity. Traditional monitoring based on body mass index (BMI, kg/m^2^) and weight primarily captures quantitative recovery, failing to reflect early qualitative metabolic and functional restoration. This study evaluated the longitudinal associations between improvements in physical performance and metabolic and structural recovery during intensive rehabilitation. **Methods:** A prospective longitudinal study was conducted on 21 AN patients undergoing a four-month intensive nutritional and functional rehabilitation program at Villa Miralago. Anthropometry, BIVA-derived parameters, indirect calorimetry and physical performance tests were assessed at baseline (T0), 2 months (T1) and 4 months (T2). Longitudinal associations were explored using Generalized Estimating Equations (GEE). **Results:** Improvements in functional performance (KPI-SUSS) were significantly associated with metabolic recovery, with a positive association with resting metabolic rate (β = +0.3635; *p* = 0.0088), indicating early metabolic reactivation before full structural reconstruction. Grip strength (KPI-grip) was significantly associated with cellular integrity (Xc β = +0.4129; *p* = 0.0088) and with favorable fluid redistribution (↑ intracellular water percentage (ICWp), ↓ extracellular water percentage (ECWp)). Structural recovery markers (Fat-Free Mass (FFM, kg), Fat-Free Mass Index (FFMI, kg/m^2^), BCM) increased significantly over time, confirming a time-dependent restoration of metabolically active mass. Fat mass (FM, kg) and fat mass percentage (FMp) were positively associated with functional improvement, although this effect attenuated longitudinally. These findings support strength performance as a sensitive functional indicator longitudinally associated with qualitative metabolic and cellular recovery. **Conclusions:** Muscle strength and functional performance tests are simple, non-invasive and cost-effective tools longitudinally associated with metabolic and structural normalization in AN. Their integration into clinical practice may enable the monitoring of meaningful recovery, personalized rehabilitation strategies and improving long-term outcomes.

## 1. Introduction

Anorexia nervosa (AN) is a severe and complex psychiatric disorder characterized by persistent and voluntary restriction in energy intake, driven by an intense fear of weight gain and distorted body image perception [1]. Beyond its psychopathological components, AN represents a state of chronic protein–energy malnutrition with profound consequences for metabolic status, body composition, and physical functioning [2,3].

Prolonged energy deprivation leads to a disproportionate depletion of body compartments, with reductions in fat mass (FM) and Fat-Free Mass (FFM), including Body Cell Mass (BCM), which represents the metabolically active component of body tissues [4]. These structural alterations are associated with physiological adaptations to starvation, including a decrease in resting metabolic rate (RMR, kcal/day), impaired muscle function, and a marked decline in strength and physical performance [5,6].

Recent epidemiological evidence from European countries indicates that anorexia nervosa represents a rapidly growing public health concern across Europe. The Global Burden of Disease Study 2021 documented a substantial increase in disease burden between 1990 and 2021, with Western Europe among the most affected regions and a faster rate of increase among males [7]. Consistent with these trends, large national registry studies from Denmark and France have reported rising incidence, greater clinical severity, and a marked increase during and after the COVID-19 pandemic, particularly among younger age groups [8,9]. Together, these data provide a strong rationale for improving early detection and dynamic monitoring of recovery during nutritional rehabilitation.

In the treatment of AN, weight restoration is a necessary but not sufficient therapeutic goal. It is widely recognized that body weight gain, assessed through traditional anthropometric indices such as body mass index (BMI), does not necessarily reflect the qualitative recovery of body compartments of the restoration of functional and metabolic capacity [10,11]. This highlights the need for more sensitive and dynamic tools to monitor physiological recovery during nutritional rehabilitation.

Bioelectrical Impedance Vector Analysis (BIVA) is an advanced, non-invasive technique for assessing body composition, hydration status, and cellular integrity, overcoming some of the limitations associated with predictive equations [12,13]. Parameters such as resistance (Rx, Ω), reactance (Xc, Ω), and phase angle (PA, Ω) provide indirect yet clinically meaningful information on tissue quality, fluid distribution, and cellular nutritional status, making BIVA particularly suitable for complex clinical populations such as individuals with AN [14].

Concurrently, functional assessment of muscle strength has gained increasing relevance as an integrated biomarker of nutritional status and metabolic health. Simple and reproducible tests such as handgrip strength (HGS, kg) and lower-limb physical performance measures (e.g., Sit-Up Test, Squat-Stand Test and Chair-Stand Test) have been proposed as prognostic markers of clinical outcomes, functional recovery, and mortality in various clinical populations, including individuals with AN [15,16,17,18,19,20]. Muscle strength reflects not only muscle quantity but also muscle quality, integrating neuromuscular and metabolic components that may recover earlier than structural tissue mass.

Despite the increasing interest in integrating structural, metabolic, and functional markers in AN monitoring, the dynamic interactions between these variables during nutritional rehabilitation remain poorly explored, particularly in longitudinal studies conducted in intensive treatment settings. It is still unclear whether early changes in physical performance are longitudinally associated with subsequent changes in body composition, fluid distribution, and resting metabolic function.

Therefore, the aim of this study was to investigate, in a cohort of patients with AN undergoing intensive nutritional rehabilitation, the longitudinal relationships between bioimpedance-derived markers, metabolic parameters, and muscle strength indicators using a Generalized Estimating Equations (GEE) statistical approach. We hypothesized that functional recovery, expressed through muscle strength performance, is longitudinally associated with qualitative changes in body compartments and metabolism, supporting its potential clinical relevance as an indicator of physiological recovery beyond weight restoration alone.

## 2. Materials and Methods

### 2.1. Study Design and Setting

The study followed a non-interventional observational longitudinal design, conducted at Villa Miralago (VM), a specialized residential center for the treatment of Eating Disorders (ED), Cuasso al Monte, Varese, Italy. Data were collected during routine clinical practice in patients undergoing intensive nutritional rehabilitation, without introducing any additional procedures for research purposes. A total of 21 patients diagnosed with AN were monitored over a hospitalization period of at least four months, until May 2025. Written informed consent for the use of anonymized clinical data for research purposes was obtained from all participants (and/or legal guardians).

Eligibility criteria included: (1) confirmed diagnosis of AN according to the DSM-5 TR, verified by a psychiatrist [1,21]; (2) admission and hospitalization at VM; and (3) age > 15 years. Exclusion criteria involved: (1) presence of an ED diagnosis different from AN (e.g., bulimia nervosa or binge eating disorder); (2) age under 15 years and (3) hospitalizations shorter than four months. Additionally, individuals meeting inclusion requirements were not considered eligible if clinical (e.g., infections, inflammatory states, or other clinically unstable conditions) or psychopathological conditions prevented adequate monitoring of study variables. Patients with unstable psychiatric comorbidities or medical conditions incompatible with the nutritional rehabilitation process were excluded, consistent with VM’s admission criteria.

### 2.2. Characteristics of the Study Sample

The study began with the recruitment of 31 patients (including adults and adolescents aged over 15 years). Subjects were included because they met the inclusion criteria, meaning they had a diagnosis of AN and were hospitalized at VM, where they were undergoing intensive nutritional rehabilitation.

However, the final analyzed sample consisted of 21 patients, 20 affected by AN restricting subtype and only 1 by AN binge–purge subtype. It is important to note that, within the intensive residential rehabilitation setting of Villa Miralago, binge–purge behaviors are closely monitored and managed through a structured multidisciplinary treatment program. A drop-out of 10 patients occurred due to self-discharge or early discharge from the VM Community during the study period. The definitive sample of 21 patients who completed the measurements at the three time points (T0, T1, and T2) included both sexes, although a clear majority (20 patients) were female (only 1 patient was male). The mean age was 24 years (from 15 to 45). These demographic characteristics are consistent with typical data from the population treated in specialized AN care setting.

### 2.3. Nutritional and Functional Rehabilitation Intervention

#### 2.3.1. Nutritional Rehabilitation

Patients admitted to VM followed an integrated, multidisciplinary and analytically oriented clinical model. The approach addressed the complexity of AN by acting simultaneously on metabolic, nutritional, emotional and behavioral processes, moving beyond symptom control toward a qualitative recovery of the patient.

The intensive treatment provided to hospitalized patients with AN followed an integrated, multidisciplinary and analytically oriented clinical model adopted at VM. This therapeutic approach aimed at comprehensive recovery of the patient, combining metabolic and nutritional rehabilitation with psychological and emotional processing, moving beyond symptom control and focusing on qualitative restoration rather than weight gain alone.

Nutritional intervention was central to rehabilitation and was monitored using objective physiological parameters.

Individualized dietary plans were structured to promote weight restoration and metabolic normalization through dynamic caloric–protein adjustments guided by clinical markers [4]. The objective was to support cellular regeneration and preserve BCM while avoiding disproportionate fat regain. Objective monitoring included periodic anthropometry and BIVA to evaluate body composition, hydration status and cellular integrity, and Indirect Calorimetry (IC) to assess RMR and guide nutritional calibration.

#### 2.3.2. Functional Rehabilitation

Given that AN induces muscle weakness and qualitative alterations in muscle tissue, functional rehabilitation was implemented through structured, supervised physical activity—including progressive resistance training—to improve muscle quality and strength. This intervention also contributed to emotional regulation, reducing anxiety, depressive symptoms, and compulsive hyperactivity. Training plans were individualized based on clinical status (e.g., malnutrition severity, cardiovascular safety), and muscle strength was continuously monitored as a critical predictive indicator of recovery and as a dynamic tool for therapeutic adaptation. Functional recovery was objectively evaluated through the handgrip strength test (HGS) and the Sit-Up Squat Stand (SUSS) functional test battery, assessing upper- and lower-limb strength, muscle power, and overall physical performance.

### 2.4. Study Timeline and Data Collection Protocol

The study was conducted longitudinally over a four-month period for each participant. Data collection was systematically scheduled at three consecutive time points: Baseline (T0), T1 (two months post-T0), and T2 (two months post-T1). To account for clinical variables and organizational needs, assessments were performed within a flexible time window of a few days around the scheduled dates. All patients had complete data at all time points; therefore, no imputation or missing-data handling procedures were necessary. Variables were categorized across these three measurements, starting with time-invariant covariates such as confirmed AN diagnosis [1], sex, age, height, and prevalent symptomatology.

### 2.5. Instrumentation and Measurement Techniques

#### 2.5.1. Anthropometric Measurements

Body weight was measured using a Kern MPB 300K100 professional scale (KERN & Sohn GmbH, Balingen, Germany; Class III medical device, 0.1 kg resolution), between 7:30 and 8:30 AM, with patients fasting and wearing light clothing. The Hold function was used to fix the weight value once stabilized, allowing accurate and reliable measurements.

Height was measured using the wall-mounted mechanical Wunder W030299 stadiometer (Wunder S.A.S., Trezzo sull’Adda, Milan, Italy).

#### 2.5.2. Bioimpedance Vector Analysis (BIVA)

BIVA was used to assess body composition, cellular integrity and hydration status. A phase-sensitive impedance analyzer (Akern BIA 101 BIVA PRO, 50 kHz; Akern S.r.l., Florence, Italy) was employed with low-intensity alternating current. Measurements were performed using the classical tetrapolar electrode configuration on the right side of the body, with patients lying supine. After at least 10 min of rest to allow fluid stabilization, two current electrodes were placed on hand and foot, and two sensing electrodes on wrist and ankle.

Raw impedance values (Rx, Xc) were recorded and used to derive PA and estimate body compartments including FFM, FM, total body water (TBW, L), extracellular water (ECW, L), intracellular water (ICW, L) and BCM, using validated predictive equations [12,13]. Data processing and vector interpretation were performed using Akern BodyGram PRO software (version HBO 3.0.33, Akern S.r.l., Florence, Italy). In the present study, bioimpedance-derived parameters were analyzed as continuous numerical variables for association analyses. The BIVA framework was used to support the physiological interpretation of these parameters.

#### 2.5.3. Indirect Calorimetry (IC)

IC, considered the gold standard for metabolic assessment, was used to measure RMR. Measurements were performed using the Vyntus™ CPX system (CareFusion/VIASYS Healthcare, Hoechberg, Germany) in canopy mode [22,23]. The system continuously analyzed oxygen consumption (VO_2_) and carbon dioxide production (VCO_2_) via electrochemical gas analyzers and turbine flow sensors. Steady state during indirect calorimetry was defined as a period of at least 5 consecutive minutes during which VO_2_ and VCO_2_ varied by less than 10% and RQ by less than 5%. Resting energy expenditure was calculated exclusively from the steady-state period.

Assessments were carried out under standardized conditions: patients were tested in the morning after an overnight fast (≥10 h), in supine position, awake but relaxed, in a thermoneutral room (22–24 °C). After a 10 min acclimatization period, gas exchange was recorded for 20 min, and only data obtained during a stable metabolic plateau were used for analysis. RMR was calculated using the Weir equation and without normalization for Fat-Free Mass. This approach was chosen because FFM in AN may not reflect metabolically active tissue uniformly, and absolute RMR is more informative for clinical management. The respiratory exchange ratio (RER = VCO_2_/VO_2_) was determined to evaluate substrate utilization and was analyzed as an absolute value (kcal/day) and values < 0.67 or >1.00 were excluded as physiologically invalid. Data acquisition and computation were performed using SentrySuite software (version 2.21, CareFusion/VIASYS Healthcare, Hoechberg, Germany).

#### 2.5.4. Muscle Strength and Functional Performance Tests

##### Handgrip Strength Test (HGS)

Upper-limb muscle strength was evaluated using the HGS, measured with a Baseline^®^ Hydraulic Hand Dynamometer (Fabrication Enterprises Inc., White Plains, NY, USA).

This test assesses maximal voluntary isometric contraction of the forearm and hand flexor muscles, serving as a functional marker of neuromuscular performance and physical risk in AN [15,24,25,26].

Participants were seated, with shoulders adducted, elbows flexed at 90°, and wrists in neutral position, following standardized guidelines that ensure high inter- and intra-test reliability, as recommended by the American Society of Hand Therapists (ASHT) [27]. They were instructed to squeeze the dynamometer as hard as possible for 3–5 s. Three trials were performed for each hand, alternating sides with ~30 s rest to minimize fatigue [19]. The highest value (kg) among all attempts was retained for analysis.

##### SUSS Test Battery

The SUSS Test is a rapid functional test battery specifically recommended for assessing muscle power and physical status in hospitalized patients with AN, according to MARSIPAN guidelines [19]. This assessment reflects lower-limb power and provides a clinically relevant risk indicator, as strength-based tests are difficult for patients to falsify when attempting to mask physical deterioration. Both SUSS and handgrip strength are clinically relevant because they assess muscle power and physical risk through performance-based measures that are difficult to falsify and provide information that is not fully captured by body weight or BMI alone [19].

The battery included three subtests:

- Sit-Up Test

The Sit-Up Test evaluates trunk flexor strength and control of abdominal and hip-flexor muscles by assessing the patient’s ability to rise from a supine to a seated position [16]. The examiner observes whether the patient recruits the arms for assistance, the speed of movement, and the quality of execution. Performance is scored using a 4-point ordinal scale: 0 = unable to perform (typically in severe AN); 1 = able to rise only using arms/legs for leverage; 2 = rises with difficulty, using limbs for balance; 3 = rises smoothly without assistance (normal performance).

- Squat-Stand Test

The Squat-Stand Test assesses lower-limb strength, particularly hip flexor and extensor muscle function. The patient begins in a squat position (knees flexed) and is asked to stand up without using the hands or external support. Performance is rated using the same 0–3 ordinal scale applied in the Sit-Up Test [19].

- Chair-Stand Test

The Chair-Stand Test measures lower-limb muscle power and endurance, evaluating the patient’s ability to rise from a chair—a functional daily activity also referred to as sit-to-stand (STS). Muscle power has been shown to be a stronger predictor of functional limitation than muscle strength alone or gait speed [16].

For this test, the patient repeatedly stands up and sits down using only the legs (arms crossed over the chest), keeping the trunk upright. A stopwatch and a standardized chair (backrest, height ~43–44 cm) placed against a wall for stability are required. The outcome variable is the number of full sit-to-stand repetitions completed in 30 s.

Together, HGS and SUSS-derived measures provided objective indicators of muscle strength, functional performance and physical recovery, complementary to body composition and metabolic parameters.

### 2.6. Statistical Analysis

#### 2.6.1. Data Structure

The analysis was performed on a longitudinal dataset consisting of 21 patients, each evaluated at three consecutive time points (T0, T1, T2), resulting in a total of 63 observations.

Variables were classified into:

Response variables (markers): anthropometric (BMI), bioimpedance-derived parameters (Rx, Xc, PA), body composition indices (FM, fat mass percentage (FMp), FFM, Fat-Free Mass percentage (FFMp), Fat-Free Mass Index (FFMI), BCM, TBW, total body water percentage (TBWp), ICW, intracellular water percentage (ICWp), ECW, extracellular water percentage (ECWp)), and metabolic variables (RMR).

Predictor variables (muscle strength): physical performance outcomes, including SUSS functional tests (Sit-Up, Squat-Stand, Chair-Stand), and HGS Test results. For handgrip assessment, only values obtained from the dominant hand were included in inferential models to ensure consistency.

#### 2.6.2. Normalization and Performance Indices

To ensure comparability between body markers and strength outcomes, all variables were standardized prior to statistical modeling.

All physiological markers (response variables) were normalized using a Min–Max scaling method, rescaling each variable to the interval 0–1 according to: (x-min(x))/[max(x)-min(x)].

Raw strength test scores were summarized into two normalized Key Performance Indicators (KPIs):

KPI Handgrip (KPI grip) (dominant hand strength values)

KPI SUSS (Sit-Up + Squat-Stand + Chair-Stand)

KPI scores were computed using a z-score-based aggregation, integrating individual scores relative to their mean and variance distributions.

#### 2.6.3. Inferential Modeling

Given the correlated longitudinal structure and inter-individual variability, inferential analysis was conducted using GEE [28,29].

For each normalized outcome variable (Y_it_), a GEE model assuming a Gaussian family with identity link function was estimated. Fixed effects included time, KPI-SUSS, KPI-handgrip, and their interaction terms:

Y_it_ = β_0_ + β_1_ time_it_ + β_2_ KPI_SUSS_it_ + β_3_ KPI_grip_it_ + β_4_ (time_it_ × KPI_SUSS_it_) + β_5_ (time_it_ × KPI_grip_it_) +ε_it_

where i = 1,…,21 (patients) and t = 1,…,3 (timepoints).

The error term ε_it_ within subjects followed an AR(1) correlation structure, reflecting stronger correlation between closer temporal measurements.

Statistical significance of regression coefficients (β) was assessed using the Wald test, with α = 0.05. Effects were considered significant for *p* < 0.05.

Given the limited number of observation points, time was modeled as a linear effect.

## 3. Results

The following results were derived from the GEE model applied to the longitudinal data (63 observations from 21 patients) to quantify the association between normalized body markers and strength indicators (normalized KPI SUSS and normalized KPI grip), considering evolution over time. The Estimate values (GEE regression coefficients β) indicate the direction and magnitude of the relationship, while a *p*-value < 0.05 confirms statistical significance.

### Association Between Body Markers and Strength Indicators

Normalized BMI was significantly influenced exclusively by time (*p*-value = 0.0495), with a positive Estimate of 0.2336 (Table 1-A). Practically, this result indicates a significant increase in normalized BMI during the rehabilitation process. The terms related to the strength KPIs (normalized KPI SUSS and normalized KPI grip) and temporal interactions did not show significant associations with normalized BMI (*p*-value normalized KPI SUSS = 0.1083, *p*-value normalized KPI grip = 0.7553, *p*-value time × normalized KPI SUSS = 0.3223, *p*-value time × normalized KPI grip = 0.8735). This suggests that the body mass index is not significantly correlated with the strength indices, nor with their temporal dynamics.

In Table 1-B the GEE analysis does not show significant direct associations between strength KPIs and normalized Rx (*p*-value normalized KPI SUSS = 0.2659, *p*-value normalized KPI grip = 0.2237), nor a significant variation in Rx over time (*p*-value = 0.4892). Accordingly, normalized electrical resistance does not show statistically significant changes over the rehabilitation period (from T0 to T2) and is not significantly associated with strength indicators. No statistically significant relationship was found for the time × KPI grip term either (*p*-value = 0.3060). The only term that achieved statistical significance was normalized time × KPI SUSS (*p*-value = 0.0314), with a negative Estimate of −0.229. This negative coefficient indicates that the strength of the association between electrical resistance and SUSS functional performance significantly decreased with the progress of recovery time.

Normalized Xc showed a statistically significant positive association with normalized KPI grip (*p*-value = 0.0088, Estimate = 0.4129), as well as significant interaction with time (normalized time × KPI grip: *p*-value = 0.0401; Estimate = −0.2030) (Table 1-C). These findings indicate that higher grip strength values were associated with higher normalized reactance values, while the magnitude of this positive association decreased over time. No significant association with Xc was found for all other terms: *p*-value Time = 0.1905, *p*-value normalized KPI SUSS = 0.5275, *p*-value normalized time × KPI SUSS = 0.1715.

Regarding normalized PA, no statistically significant associations were observed for any of the examined covariates or interaction terms (Table 1-D), as none reached the statistical significance threshold (α = 0.05). All coefficients are reported below, although non-significant: the effect of time is Estimate = 0.1446 (*p*-value = 0.2837); normalized KPI SUSS has a negative Estimate (−0.1299), with *p*-value = 0.4016; normalized KPI grip has the highest Estimate (0.3046), with a *p*-value = 0.0650; the interaction normalized time × KPI SUSS has a negative Estimate (−0.0651), with *p*-value = 0.6097; the interaction normalized time × KPI grip has a negative Estimate (−0.1958), with *p*-value = 0.1050. Practically, the results suggest that the normalized PA does not show statistically significant longitudinal associations with strength performance within the observed period.

As shown in Table 1-E, normalized FM was significantly associated with: time (*p*-value = 0.0091), with a positive Estimate of 0.2504 (practically, normalized fat mass significantly increased during the rehabilitation period); normalized KPI SUSS (*p*-value = 0.0012), with a positive Estimate of 0.4822. Practically, a positive longitudinal association was observed between SUSS performance and normalized fat mass. However, the significant negative interaction between time and KPI SUSS (Estimate = −0.3765; *p* = 0.0041) indicates that the strength of this association decreased over the observation period, reflecting a progressive attenuation of their co-variation during nutritional rehabilitation. Practically, the strength of the positive association between FM and SUSS performance significantly attenuates with the progress of the observation time. No significant direct effects of normalized KPI grip (*p*-value = 0.2972) or normalized time × KPI grip (*p*-value = 0.6194) were found on FM.

The results for normalized FMp are consistent with those for FM (Table 1-F): significant association with time (*p*-value = 0.0460, Estimate = 0.1907), normalized KPI SUSS (*p*-value = 0.0218, Estimate = 0.3759), and normalized time × KPI SUSS (*p*-value = 0.0142, Estimate = −0.3029). No significant direct effects of normalized KPI grip (*p*-value = 0.2050) or normalized time × KPI grip (*p*-value = 0.8701) were found on FMp.

The GEE analysis in Table 1-G shows a statistically significant positive association (*p*-value = 0.0127) between normalized FFM and time, with a positive Estimate (0.2362), indicating that FFM increased during the rehabilitation period. No statistically significant association was found between normalized FFM and the normalized physical strength indicators (*p*-value normalized KPI SUSS = 0.5385, *p*-value normalized KPI grip = 0.5195). Likewise, no significant association was found for the interactions normalized time × KPI SUSS (*p*-value = 0.2429) and normalized time × KPI grip (*p*-value = 0.3522), suggesting no statistically significant longitudinal associations between FFM and physical performance within the observed period.

Table 1-H shows that normalized FFMp was significantly associated with: time (*p*-value = 0.0350), with a negative Estimate of −0.2014 (indicating that normalized FFMp significantly decreased during the rehabilitation period); normalized KPI SUSS (*p*-value = 0.0007), with a negative Estimate of −0.5168 (this means that SUSS performance and normalized FFMp show a negative longitudinal association); normalized time × KPI SUSS (*p*-value = 0.0011), with a positive Estimate of 0.3937, indicating that the magnitude of the negative association between FFMp and KPI SUSS decreased over time. In practical terms, the strength of the negative association between FFMp and SUSS performance significantly decreases over time. No significant direct effects of normalized KPI grip (*p*-value = 0.3659) or normalized time × KPI grip (*p*-value = 0.9099) were found on FFMp.

The results for normalized FFMI are consistent with those relative to FFM (Table 1-I): the effect of time was highly significant (*p*-value = 0), with a strong positive Estimate (0.4807), indicating a constant and marked increase in FFMI during the rehabilitation period. No statistically significant relationship was found between normalized FFMI and the normalized physical strength indicators (*p*-value normalized KPI SUSS = 0.9783, *p*-value normalized KPI grip = 0.9154). Similarly, no significant evidence was found for the interactions normalized time × KPI SUSS (*p*-value = 0.4505) and normalized time KPI grip (*p*-value = 0.9154), suggesting no statistically significant longitudinal associations between FFMI and physical performance, and that this condition does not change over time.

Normalized BCM was significantly associated exclusively with time (*p*-value = 0.0106), with a positive Estimate of 0.2935 (Table 1-J), indicating that normalized BCM significantly increased during the rehabilitation process. The terms related to the strength KPIs (normalized KPI SUSS and normalized KPI grip) and temporal interactions did not show significant associations with normalized BCM (*p*-value normalized KPI SUSS = 0.7111, *p*-value normalized KPI grip = 0.5372, *p*-value normalized time × KPI SUSS = 0.7311, *p*-value normalized time × KPI grip = 0.0771).

No statistically significant associations were observed for any of the examined covariates or interaction terms (Table 1-K) with normalized TBW, as none reached the statistical significance threshold (α = 0.05). All coefficients are reported below: the effect of time is Estimate = 0.0059 (*p*-value = 0.9564); normalized KPI SUSS has a negative Estimate (−0.0980), with *p*-value = 0.5409; normalized KPI grip has a negative Estimate (−0.2437), with *p*-value = 0.0724; the interaction normalized time × KPI SUSS has an Estimate of 0.1369 (*p*-value = 0.1944); the interaction normalized time × KPI grip has an Estimate of 0.1536, with *p*-value = 0.1229. Normalized total body water did not show statistically significant longitudinal changes or associations with physical strength within the observed period.

Unlike absolute TBW, normalized TBWp presents three statistically significant associations (Table 1-L): with time (*p*-value = 0.0151; Estimate = −0.2538) with normalized KPI SUSS (*p*-value = 0.0127; Estimate = −0.3951), and with the interaction normalized time × KPI SUSS (*p*-value = 0.0236; Estimate = 0.2808). These findings indicate that: normalized TBWp significantly decreased during the rehabilitation time; a negative longitudinal association between TBWp and SUSS performance, the magnitude of which decreased over time. No statistically significant associations were observed for normalized KPI grip (*p*-value = 0.0914) or normalized time × KPI grip (*p*-value = 0.5581) with TBWp.

In the analysis of normalized intracellular water (ICW), no statistically significant associations were observed for any of the examined covariates or interaction terms (Table 1-M): all terms were above the statistical significance threshold (α = 0.05). The effect of time shows an Estimate value of 0.0941 (*p*-value = 0.4253). Normalized KPI SUSS has an Estimate of −0.0442 (*p*-value = 0.8086). Normalized KPI grip has Estimate = −0.1222 (*p*-value = 0.3853). The interaction normalized time × KPI SUSS has Estimate = 0.1129 and *p*-value = 0.3233. The interaction normalized time × KPI grip has Estimate = 0.0593 and *p*-value = 0.6000. Practically, ICW does not show significant longitudinal associations with time, strength indicators, or their temporal interactions.

The normalized intracellular water percentage (ICWp) presents a single statistically significant association with grip strength (normalized KPI grip), with *p*-value = 0.043 and a positive Estimate of 0.3121 (Table 1-N). These findings indicate a positive longitudinal association between grip strength and normalized intracellular water percentage. The terms related to time, normalized KPI SUSS, and temporal interactions did not show significant associations with normalized ICWp (*p*-value time = 0.3182, normalized KPI SUSS = 0.4877, *p*-value normalized time × KPI SUSS = 0.7445, *p*-value normalized time × KPI grip = 0.0142).

Normalized ECW presented a significant association with grip strength, as well as with time (Table 1-O). The effect of normalized KPI grip has a *p*-value of 0.0143, with a negative Estimate of −0.4110, while the interaction effect normalized time × KPI grip has a *p*-value of 0.0142 and a positive Estimate of 0.2286. These findings indicate an inverse longitudinal association between grip strength and normalized extracellular water; thus, an increase in grip strength is associated with a significant decrease in normalized extracellular water, and the strength of this negative association significantly decreases over time. The terms related to time, normalized KPI SUSS, and normalized time KPI SUSS did not show significant associations with normalized ECW (*p*-value time = 0.3743, normalized KPI SUSS = 0.8233, *p*-value normalized time × KPI SUSS = 0.8233).

The results for normalized ECWp are consistent with those observed for absolute normalized ECW with respect to grip strength (Table 1-P): normalized KPI grip showed a statistically significant association (*p*-value = 0.0486), with a negative Estimate of −0.3050. However, unlike absolute ECW, no statistically significant association was found for the interaction between ECWp and normalized time × KPI grip (*p*-value = 0.1699). Likewise, no statistically significant associations were observed for the interactions between ECWp and time (*p*-value = 0.3269), normalized KPI SUSS (*p*-value = 0.5306), and normalized time × KPI SUSS (*p*-value = 0.8775).

Normalized RMR presented a statistically significant positive association with normalized KPI SUSS (*p*-value = 0.0088, Estimate = 0.3635), as shown in Table 1-Q. Practically, this association indicates that higher SUSS performance scores were associated with higher normalized resting metabolic rate at the same observation time. Conversely, no statistically significant associations were found for time (*p*-value = 0.4069), normalized KPI grip (*p*-value = 0.5077), or normalized time × KPI SUSS (*p*-value = 0.2407) or normalized time × KPI grip (*p*-value = 0.6111). This suggests that the magnitude of the association between RMR and physical performance does not significantly change during the observation time.

For clarity, effect sizes with corresponding 95% confidence intervals for significant associations are summarized in Table 2.

## 4. Discussion

AN is a severe psychiatric disorder characterized by the voluntary restriction of energy intake driven by an intense fear of weight gain [1,2]. This chronic and severe protein–energy malnutrition leads to a disproportionate depletion of body compartments, severely reducing both FM and FFM, including BCM [4,30]. These structural losses, combined with the physiological adaptations to prolonged starvation, result in inevitable functional impairment and a significant reduction in RMR [31,32].

The primary objective of this study was to identify and quantify the dynamic relationships and longitudinal associations among bioimpedance, metabolic, and physical performance markers in this clinical context. Previous analyses of comparable datasets have suggested that weight and physical strength recovery tended to manifest earlier than the structural recovery of FFM [10]. To capture the complex dynamics of recovery and account for the notable inter-individual heterogeneity among patients, the GEE model was employed. This approach allowed us to investigate whether physical performance test results (Key Performance Indicators, KPI) are longitudinally associated with variations in bioimpedance and metabolic markers, supporting their potential clinical relevance as functional indicators tracking physiological recovery.

### 4.1. Structural Recovery over Time

The GEE model results showed that body compartment measures and body weight exhibited statistically significant longitudinal changes associated with time.

Anthropometric and FFM Markers

Normalized BMI was significantly associated exclusively with the time factor (Estimate = 0.2336, *p* = 0.0495). Similarly, absolute mass markers showed statistically significant positive associations with: FFM (Estimate = 0.2362, *p* = 0.0127), FFMI (Estimate = 0.4807, *p* = 0), and BCM (Estimate = 0.2935, *p* = 0.0106). This positive relationship indicates that these parameters increased over the observed nutritional rehabilitation period, consistent with the recognized importance of FFMI in assessing nutritional risk [33].

Although BMI recovery was statistically significant, its marginal *p*-value (*p* = 0.0495) and its lack of association with strength indices underscore its poor robustness as an indicator of qualitative and functional recovery [32]. The more robust, exclusive longitudinal associations of FFM, FFMI, and BCM with time confirm that the rebuilding of metabolically active mass is a gradual process that unfolds only over the observation period.

FM and Percentage Composition

An increase in FM, both absolute and percentage, is an expected and necessary outcome for functional and endocrine recovery [2,34]. The GEE analysis confirmed that the increase in FM (Estimate = 0.2504, *p* = 0.0091) and FMp (Estimate = 0.1907, *p* = 0.0460) was positively and significantly associated with observation time.

Conversely, FFMp (Estimate = −0.2014; *p* = 0.0350) and TBWp (Estimate = −0.2538; *p* = 0.0151) decreased significantly over time. This trend is not unfavorable but is consistent with the progressive normalization of body composition. As treatment advances, the recovering weight includes a necessary increase in FM. Since the expansion of the adipose compartment is associated with a reduction in the relative fraction of FFM and TBWp (given FFM is the primary water compartment), this proportional effect is consistent with compositional normalization, despite increasing absolute FFM values.

### 4.2. Functional Strength (KPI SUSS) in Relation to Metabolic and Body Recomposition Markers

The GEE analysis successfully identified a longitudinal association between functional recovery and physiological normalization, recognizing that muscle weakness involves both quantitative FFM loss and qualitative skeletal muscle alterations [35]. The Key Performance Indicators related to functional strength (KPI SUSS), which include the muscle power measurement of the Chair-Stand Test [16], revealed significant longitudinal associations with metabolic parameters and body recomposition dynamics.

RMR was positively and significantly associated with KPI SUSS (Estimate = 0.3635, *p* = 0.0088), indicating a longitudinal association between functional strength and metabolic recovery. These findings are consistent with previous evidence [10] suggesting that neuromuscular adaptation and strength gains may occur earlier than full tissue reconstruction and may be temporally aligned with early changes in resting energy expenditure.

FM/FMp: KPI SUSS showed a positive and significant association with FM (Estimate = 0.4822, *p* = 0.0012) and FMp (Estimate = 0.3759, *p* = 0.0218), indicating that functional performance and fat mass parameters co-vary longitudinally during the nutritional rehabilitation process.

However, the present study was not designed to disentangle independent or mechanistic effects of specific body composition compartments on functional outcomes. Rather, its aim was to longitudinally describe the multidimensional process of physical recovery in anorexia nervosa, in which changes in body composition and functional performance evolve concurrently during nutritional rehabilitation. Accordingly, the observed associations between functional performance and FM parameters should be interpreted as co-variation during weight restoration rather than as evidence of a direct or specific role of fat mass in functional improvement.

It is important to clarify that functional performance tests, including the SUSS battery and handgrip strength, are not intended as surrogate markers of body weight, BMI, or body composition changes. Rather, they are clinically established measures of neuromuscular function and muscle power, which are used in anorexia nervosa precisely because they capture aspects of physical risk and functional impairment that are not fully explained by anthropometric or body composition measures.

Indeed, as reported by Etemadi et al. [19], SUSS tests and handgrip strength represent valid and reliable measures of muscle power in anorexia nervosa and are recommended for clinical risk assessment, given that weight and BMI alone may be misleading indicators of functional status.

Therefore, the observed longitudinal associations between functional performance and FM parameters should not be interpreted as indicating a specific or mechanistic role of FM in functional improvement. Rather, they likely reflect the global nutritional rehabilitation process, in which changes in body composition and neuromuscular function occur concurrently.

Importantly, improvements in functional performance may occur independently of changes in FM, FFM, or BMI, and should be interpreted as reflecting neuromuscular and functional recovery rather than simple anthropometric gain.

This interpretation is further supported by evidence showing that muscle function responds earlier to nutritional deprivation and repletion than body composition parameters and may improve independently of changes in muscle mass or Fat-Free Mass, particularly in malnutrition and anorexia nervosa [15].

In line with this, SUSS and handgrip strength are recommended components of clinical risk assessment in anorexia nervosa (MARSIPAN guidelines) and are used precisely because body weight and BMI may be unreliable or falsified, highlighting that functional performance captures a dimension of physical risk that is not reducible to anthropometric measures [19].

Temporal Mitigation of FM Accumulation: a clinically relevant longitudinal pattern was observed, characterized by a negative and significant interaction term time × KPI SUSS on FM (Estimate = −0.3765, *p* = 0.0041) and FMp (Estimate = −0.3029, *p* = 0.0142). This suggests that the strength of the positive association between functional performance and fat mass accumulation decreased over observation time, consistent with a progressive modulation of body recomposition patterns.

FFMp: KPI SUSS showed a significant negative association with FFMp (Estimate = −0.5168, *p* = 0.0007). This inverse association is an expected clinical consequence of recovery. As the patient recovers weight, and both FFM (in absolute terms) and FM increase, the relative percentage of FFM mathematically decreases due to the proportional FM gain (which has negligible protein/water content) [32]. The significant positive interaction term time × KPI SUSS (Estimate = 0.3937, *p* = 0.0011) indicates that the magnitude of this inverse association decreased over time, consistent with progressive compositional stabilization.

TBWp: KPI SUSS showed a significant negative association with TBWp (Estimate = −0.3951, *p* = 0.0127). This finding is consistent with changes in body composition associated with weight restoration, as FM has a lower water content than FFM [32]. The significant positive interaction term time × KPI SUSS (Estimate = 0.2808, *p* = 0.0236) indicates that the magnitude of this negative association decreased over time, consistent with progressive fluid redistribution.

Rx: While the direct association between KPI SUSS and Rx was non-significant, the negative and significant interaction term time × KPI SUSS (Estimate = −0.229, *p* = 0.0314) is clinically notable. Since Rx is inversely proportional to TBW and FFM, its longitudinal decrease during nutritional rehabilitation (concurrent with FFM and TBW increase) shows a complex association with functional performance. The negative interaction suggests that the magnitude of the association between the structural marker (Rx) and the functional marker (KPI SUSS) decreases over time, in line with previous studies supporting the concept that functional recovery may occur earlier than structural tissue reconstruction [10].

### 4.3. Grip Strength (KPI Grip) Association with Cellular Integrity and Hydration Markers

The results from KPI grip (handgrip Test, HGS) highlighted its strong longitudinal associations with cellular markers. HGS is a widely recognized dynamic indicator of nutritional and functional status, showing consistent associations with muscular parameters like FFM and BCM [6,15,19].

Xc: KPI grip showed a positive and significant association with Xc (Estimate = 0.4129, *p* = 0.0088). Since Xc reflects cellular membrane integrity [4], this finding indicates a positive longitudinal association between grip strength and cellular membrane quality. This positive association, however, significantly attenuated over time (time × KPI grip Estimate = −0.2030; *p* = 0.0401), suggesting progressive stabilization of cellular quality.

Fluid distribution (ECW/ICW): KPI grip was inversely and significantly associated with ECW in liters (Estimate = −0.4110, *p* = 0.0143) and percentage (Estimate = −0.3050, *p* = 0.0486). This is a desirable outcome, indicating that higher grip strength values are associated with lower extracellular water levels [4]. Conversely, ICWp was positively associated with KPI grip (Estimate = 0.3121; *p* = 0.043). This pattern of associations (decreased ECW, increased ICWp) is consistent with a redistribution of body fluids toward the intracellular compartment, which has been described as a feature of qualitative recovery and active tissue regeneration [4]. The negative association with absolute ECW significantly attenuated over time (time × KPI grip Estimate = 0.2286, *p* = 0.0142).

### 4.4. Non-Significant Markers and Study Limitations

The PA, a prominent indicator of cellular integrity [36], did not show significant associations with any predictor (*p*-value = 0.0650 for KPI grip). Similarly, absolute fluid volumes (TBW and ICW in liters) were non-significant. This outcome, potentially inconsistent with some of the literature, may be attributable to the study’s methodological limitations.

Firstly, the study suffered from a small final sample size (*n* = 21) and a relatively short four-month follow-up period, which may have limited the power to detect variations in slowly evolving markers like PA.

Secondly, the context of intensive AN management is intrinsically challenging due to the psychopathology of control and illness denial [37]. This difficulty is compounded by psychiatric comorbidities [38] and behavioral relapses (e.g., excessive physical activity or purging), which frequently lead to patient dropouts and compromise physiological stability [37].

The present findings should be interpreted as exploratory and hypothesis-generating. The combination of an intensive longitudinal design and a repeated measures framework such as GEE provides a methodologically appropriate basis for identifying preliminary longitudinal associations in a clinically complex population, while acknowledging the need for confirmation in larger cohorts and the absence of formal correction for multiple comparisons.

The relatively short follow-up period precludes conclusions regarding long-term recovery trajectories or prognostic outcomes; therefore, the present findings should be interpreted as providing preliminary insight into early physiological and functional recovery dynamics and as a foundation for future studies aimed at evaluating long-term clinical relevance.

## 5. Conclusions

This longitudinal study provides novel evidence of longitudinal associations indicating that functional performance measures track with qualitative metabolic and body composition changes in patients with AN undergoing intensive nutritional rehabilitation. While structural markers such as FFM, FFMI and BCM showed a progressive, time-dependent increase, functional indicators derived from SUSS tests and handgrip strength demonstrated significant associations with fat mass redistribution, water compartment shifts and resting metabolic rate.

In particular, functional performance (KPI SUSS) was positively associated with RMR and fat mass recovery, while showing longitudinal interactions with the temporal dynamics of body recomposition, consistent with a progressive modulation of body recomposition patterns during weight regain. Grip strength (KPI grip) emerged as a relevant correlation of cellular integrity and fluid distribution, with higher values associated with increased intracellular water and reduced extracellular water, consistent with improved tissue quality and recovery of physiological hydration patterns.

Taken together, these findings indicate that muscle strength and functional performance are relevant functional indicators of physiological recovery beyond weight and BMI alone, reinforcing the limitations of relying exclusively on traditional anthropometric indices in the clinical management of AN. The integration of BIVA-derived markers, indirect calorimetry and simple, standardized functional tests (HGS, SUSS battery) provides a dynamic and clinically meaningful framework to monitor recovery trajectories.

However, the results should be interpreted in light of some limitations, including the small sample size, the single-center design, the absence of a control group and the relatively short follow-up period. Larger, multi-center studies with extended observation windows are needed to confirm these associations and to refine cut-off values and thresholds for clinical decision-making.

From a practical standpoint, our data suggest that early changes in functional strength may serve as clinically a relevant marker for monitoring the rehabilitation process in AN, and that incorporating objective functional tests alongside body composition and metabolic assessment may enhance the ability of clinicians to individualize treatment, detect qualitative recovery and support long-term prognosis.

## Figures and Tables

**Table 1:** Results of the Generalized Estimating Equations (GEE) analysis examining the relationship between body composition/metabolic parameters, KPI SUSS, KPI grip and time.

**(A) BMI**	Estimate	*p*-value	**(B) Rx**	Estimate	*p*-value	**(C) Xc**	Estimate	*p*-value
time	0.2336	0.0495 *	time	0.0756	0.4892	time	0.1639	0.1905
KPI suss	0.2374	0.1083	KPI suss	0.1768	0.2659	KPI suss	0.1031	0.5275
KPI grip	0.0478	0.7553	KPI grip	0.1707	0.2237	KPI grip	0.4129	0.0088 *
time: KPI suss	−0.1339	0.3223	time: KPI suss	−0.229	0.0314 *	time: KPI suss	−0.1805	0.1715
time: KPI grip	0.0127	0.8735	time: KPI grip	−0.0986	0.3060	time: KPI grip	−0.2030	0.0401 *
**(D) PA**	Estimate	*p*-value	**(E) FM**	Estimate	*p*-value	**(F) FMp**	Estimate	*p*-value
time	0.1446	0.2837	time	0.2504	0.0091 *	time	0.1907	0.0460 *
KPI suss	−0.1299	0.4016	KPI suss	0.4822	0.0012 *	KPI suss	0.3759	0.0218 *
KPI grip	0.3046	0.0650	KPI grip	0.1309	0.2972	KPI grip	0.1763	0.2050
time: KPI suss	−0.0651	0.6097	time: KPI suss	−0.3765	0.0041 *	Time: KPI suss	−0.3029	0.0142 *
time: KPI grip	−0.1958	0.1050	time: KPI grip	0.0542	0.6194	time: KPI grip	0.0152	0.8701
**(G) FFM**	Estimate	*p*-value	**(H) FFMp**	Estimate	*p*-value	**(I) FFMI**	Estimate	*p*-value
time	0.2362	0.0127 *	time	−0.2014	0.0350 *	time	0.4807	0 *
KPI suss	0.0743	0.5385	KPI suss	−0.5168	0.0007 *	KPI suss	−0.0018	0.9783
KPI grip	0.0761	0.5195	KPI grip	−0.1182	0.3659	KPI grip	0.0069	0.9154
time: KPI suss	0.1130	0.2429	time: KPI suss	0.3937	0.0011 *	time: KPI suss	−0.0374	0.4505
time: KPI grip	−0.0604	0.3522	time: KPI grip	−0.0111	0.9099	time: KPI grip	0.0069	0.9154
**(J) BCM**	Estimate	*p*-value	**(K) TBW**	Estimate	*p*-value	**(L) TBWp**	Estimate	*p*-value
Time	0.2935	0.0106 *	time	0.0059	0.9564	time	−0.2538	0.0151 *
KPI suss	0.0560	0.7111	KPI suss	−0.0980	0.5409	KPI suss	−0.3951	0.0127 *
KPI grip	0.0798	0.5372	KPI grip	−0.2437	0.0724	KPI grip	−0.2380	0.0914
time: KPI suss	0.0400	0.7311	time: KPI suss	0.1369	0.1944	time: KPI suss	0.2808	0.0236 *
time: KPI grip	−0.1709	0.0771	time: KPI grip	0.1536	0.1229	time: KPI grip	0.0569	0.5581
**(M) ICW**	Estimate	*p*-value	**(N) ICWp**	Estimate	*p*-value	**(O) ECW**	Estimate	*p*-value
time	0.0941	0.4253	time	0.1233	0.3182	time	−0.0974	0.3743
KPI suss	−0.0442	0.8086	KPI suss	−0.1042	0.4877	KPI suss	−0.0373	0.8233
KPI grip	−0.1222	0.3853	KPI grip	0.3121	0.043 *	KPI grip	−0.4110	0.0143 *
time: KPI suss	0.1129	0.3233	time: KPI suss	−0.0377	0.7445	time: KPI suss	0.1129	0.3740
time: KPI grip	0.0593	0.6000	time: KPI grip	0.2286	0.0142	time: KPI grip	0.2286	0.0142 *
**(P) ECWp**	Estimate	*p*-value	**(Q) RMR**	Estimate	*p*-value			
time	−0.1210	0.3269	time	0.0907	0.4069			
KPI suss	0.0944	0.5306	KPI suss	0.3635	0.0088 *			
KPI grip	−0.3050	0.0486 *	KPI grip	0.1071	0.5077			
time: KPI suss	0.0172	0.8775	time: KPI suss	−0.1345	0.2407			
time: KPI grip	0.1420	0.1699	time: KPI grip	0.0501	0.6111			

Statistically significant at *p* < 0.05 is indicated by “*”; Abbreviations: BMI: normalized body mass index; Rx: normalized electrical resistance; Xc: normalized electrical reactance; PA: normalized phase angle; FM: normalized fat mass; FMp: normalized fat mass percentage; FFM: normalized free fat mass; FFMp: normalized free fat mass percentage; FFMI: normalized Fat-Free Mass Index: BMC: normalized Body Cell Mass; TBW: normalized total body water; TBWp: normalized total body water percentage; ICW: normalized intracellular water; ICWp: normalized intracellular water percentage; ECW: normalized extracellular water; ECWp: normalized extracellular water percentage; RMR: normalized resting metabolic rate.

**Table 2:** Summary of significant associations from GEE models.

Outcome	Predictor	β	95% CI
BMI	Time	0.23	0.00 to 0.47
Rx	Time:KPI_suss	−0.23	−0.44 to −0.02
Xc	KPI grip	0.41	0.10 to 0.72
Xc	Time:KPI_grip	0.04	−0.20 to 0.10
FM	KPI SUSS	0.48	0.19 to 0.78
FM	Time:KPI SUSS	−0.38	−0.63 to −0.12
FM	Time	0.25	0.06 to 0.44
FMp	Time:KPI SUSS	−0.30	−0.55 to −0.06
FMp	KPI SUSS	0.38	0.06 to 0.70
FMp	Time	0.19	0.003 to 0.38
FFM	Time	0.24	0.05 to 0.42
FFMp	KPI SUSS	−0.52	−0.81 to −0.22
FFMp	Time:KPI_suss	0.39	0.16 to 0.63
FFMp	Time	−0.20	−0.39 to −0.01
FFMI	Time	0.48	0.40 to 0.56
BCM	Time	0.29	0.07 to 0.52
TBWp	KPI SUSS	−0.40	−0.71 to −0.09
TBWp	Time	−0.25	−0.46 to −0.05
TBWp	Time:KPI_suss	0.28	0.04 to 0.52
ICWp	KPI grip	0.31	0.01 to 0.62
ECWp	KPI grip	−0.31	−0.61 to −0.002
ECW	Time:KPI grip	0.23	0.05 to 0.41
ECW	KPI grip	−0.41	−0.74 to −0.08
RMR	KPI SUSS	0.36	0.09 to 0.64

β = regression coefficient; CI = confidence interval; GEE = Generalized Estimating Equations; KPI = Key Performance Indicator; SUSS = sit-up, squat and stand tests.

## Data Availability

The raw data supporting the conclusions of this article will be made available by the authors on request.
